# *Nicotiana benthamiana*-derived dupilumab-scFv reaches deep into the cultured human nasal epithelial cells and inhibits CCL26 expression

**DOI:** 10.1038/s41598-024-65524-0

**Published:** 2024-06-24

**Authors:** Beom Jun Kwon, Na Hyun Cho, Taeyoung Ahn, Geunah Kim, Nguyễn Thị Xuân Diệu, Woo Taek Kim, Hyung-Ju Cho, Dong Hye Seo, Joo Young Kim

**Affiliations:** 1 Department of Pharmacology and Brain Korea 21 Project for Medical Science, Yonsei University College of Medicine, 50-1 Yonsei-ro, Seodaemun-gu, Seoul, 03080 Republic of Korea; 2 Department of Otorhinolaryngology, Yonsei University College of Medicine, Seoul, Korea; 3 Department of Systems Biology, College of Life Science and Biotechnology, Yonsei University, 50 Yonsei-ro, Seodaemun-gu, Seoul, 03080 Republic of Korea; 4 Department of Pharmacognosy, Faculty of Pharmacy, University of Medicine and Pharmacy at Ho Chi Minh City, Ho Chi Minh City, Vietnam

**Keywords:** ScFv, Dupilumab, *Nicotiana benthamiana*, Human nasal epithelial cell, Non-invasive treatment, Molecular medicine, Mucosal immunology, Drug development

## Abstract

Plants offer a cost-effective and scalable pharmaceutical platform devoid of host-derived contamination risks. However, their medical application is complicated by the potential for acute allergic reactions to external proteins. Developing plant-based protein therapeutics for localized diseases with non-invasive treatment modalities may capitalize on the benefits of plant proteins while avoiding their inherent risks. Dupilumab, which is effective against a variety of allergic and autoimmune diseases but has systemic responses and injection-related side effects, may be more beneficial if delivered locally using a small biological form. In this study, we engineered a single-chain variable fragment (scFv) of dupilumab, termed Dup-scFv produced by *Nicotiana benthamiana*, and evaluated its tissue permeability and anti-inflammatory efficacy in air–liquid interface cultured human nasal epithelial cells (HNECs). Despite showing 3.67- and 17-fold lower binding affinity for IL-4Ra in surface plasmon resonance assays and cell binding assays, respectively, Dup-scFv retained most of the affinity of dupilumab, which was originally high, with a dissociation constant (KD) of 4.76 pM. In HNECs cultured at the air–liquid interface, Dup-scFv administered on the air side inhibited the inflammatory marker *CCL26* in hard-to-reach basal cells more effectively than dupilumab. In addition, Dup-scFv had an overall permeability of 0.8% across cell layers compared to undetectable levels of dupilumab. These findings suggest that plant-produced Dup-scFv can be delivered non-invasively to cultured HNESc to alleviate inflammatory signaling, providing a practical approach to utilize plant-based proteins for topical therapeutic applications.

## Introduction

Plants are an attractive manufacturing platform for biologics, offering various advantages, such as low production costs, free from host cell-derived pathogen or cell remnant contamination, and rapid production timeframe as well as scalable production size^1,2^. The first plant-produced pharmaceutical, taliglucerase alfa (ELELYSO^®^), was approved for marketing by the U.S Food and Drug Administration (FDA) to treat Gaucher disease in 2012^3^. Recently, the development of plant virus vector systems, such as the Magnicon^®^ transient vector system, has led to remarkable improvements in protein production yield and separation purity, suppressing the expression of endogenous plant proteins and expressing only introduced genes^4^. Furthermore, this system offers many astonishing features during the manufacturing phase, including rapid production cycles, high product yields, virtually unlimited scalability, and flexibility in various production plans. The utility of such plant protein expression systems almost meets the requirements for playing a foundational role in the high-value plant protein industry in the future.

ELEYSO^®^, an α-glucocerebrosidase produced by carrot callus, has been developed for the treatment of Gaucher disease and is currently in clinical use ^5^. Despite reports that the side effects of ELEYSO^®^ are not significantly different from those of Cerezyme^®^ produced by Chinese hamster ovary (CHO) cells^6^, there are still several uncertainties in plant protein production systems that need to be overcome. First, there are medical reports suggesting the risk of acute allergic reactions from intravenous injection of proteins containing plant N-glycans. Bardor et al. reported that 50% of non-allergic patients have antibodies specific to core xylose, and 25% of them have antibodies to core α (1,3)-fucose in their serum^7^. In addition, O-glycosylation in plants is quite different from that in mammals, and the full profile is not yet understood^8^. Furthermore, the post-translational modifications that occur in different species make it difficult to identify all O-glycosylation forms^9^. This uncertainty in the post-translational modification of plant proteins is a major obstacle to the development of drugs using plant proteins, given that most protein drugs are injectable.

Dupilumab (developed by Regeneron/Sanofi) is an IgG4 subclass monoclonal antibody that targets and blocks the IL-4 receptor alpha (IL-4Rα)^10^, a key regulatory cytokine in type 2 inflammation^11,12^. The drug has shown impressive therapeutic efficacy in a variety of allergic diseases, including atopic dermatitis, chronic rhinosinusitis with nasal polyps (CSwNP), and asthma. Since IL-4Rα is involved in both IL-4 and IL-13 signaling through its interaction with either IL-4Rγc or IL-13R receptors^13,14^, dupilumab, which simultaneously inhibits both cytokine responses, is highly effective in controlling type 2 inflammation^13^. However, adverse events associated with dupilumab injection, such as serum sickness syndrome and dose-dependent injection site reactions, have been sporadically reported^15–17^. Given that Type 2 inflammatory diseases are predominantly localized, there is a need for ways to mitigate injection-related side effects while maintaining the efficacy of dupilumab, and the development of formulations that can be administered non-invasively at the site of lesions is relevant. Recent antibody engineering techniques have led to the development of antibody fragments Fab, scFv, VH, and scFab that retain the antigen specificity of full-length antibodies, eliminate side effects due to immunogenicity by removing Fc, and increase penetration into target tissues by reducing size. Dupilumab works by binding to the IL4R and inhibiting IL4 binding, and is composed of the least immunogenic IgG4 Fc to prevent target-expressing cells from being killed in the process^18^. This suggests that Fc is not required for dupilumab to work. Minimizing the size of dupilumab to scFv to increase its non-invasive permeability, which is determined by the size of the material, may allow it to be used as a non-invasive route of administration, such as a nasal spray rather than an injection.

Recent single-cell RNA sequencing studies have shown that in addition to the previously known monolayer of goblet cells, multiciliated cells, and columnar cells, the lower layer of transwell cultured HNECs is composed of basal cells, whose proliferation is known to be a trigger for changes in the nasal environment, such as nasal polyp formation^14,19^. Single-cell RNA sequence results also reported that each cell expresses different inflammatory factors. MUC5A and CCL26 genes are both inflammatory marker genes whose expression is increased in sinus inflammation with increased concentrations of IL4 or IL13. However, in the case of MUC5A, it is expressed in cells that are elongated and exposed on the luminal side, such as goblet cells and columnar cells^20^. CCL26, on the other hand, is mainly expressed deep inside the cell column, where it is difficult to be exposed to the luminal side^21^. Many of these individual cellular characteristics are known to be maintained in HNECs cultured at the air–liquid interface^22^.

With the advantage of a single chain, scFv is a form of antibody that has been attempted to be produced in plants relatively early on and has been reported to be stably expressed in plants^23^. This study reports on an scFv variant of dupilumab, a plant-produced IL4 signaling inhibitor, that retains most of its affinity. The penetration and anti-inflammatory functions of air-treated Dup-scFv on air–liquid interface cultured human nasal cells were evaluated to validate the non-invasive therapeutic administration of plant-produced Dup-scFv.

## Results

### Expression and purification of the Dup-scFv with or without HDEL in *N. benthamiana*

**Figure 1:**
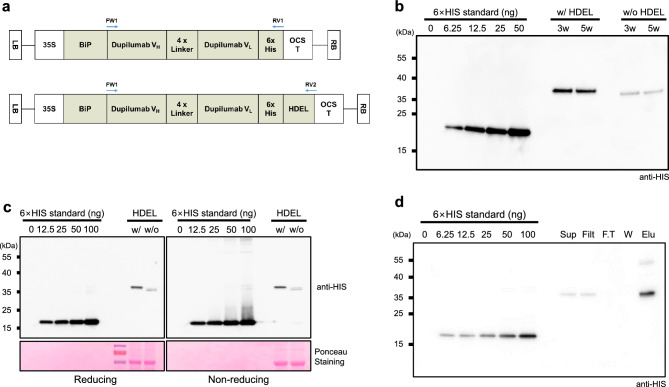
Expression and purification of the Dup-scFv in *N. benthamiana* (**a**) Schematic representation of dupilumab scFv (Dup-scFv) constructs with or without HDEL for ectopic expression in *N. benthamiana* leaves using the agrobacterium-mediated method. The sequence of dupilumab VH and VL (Supplementary Fig. 1, 2) were codon-optimized to enable efficient production of antibodies in plants. The constitutive expression was achieved using 35S promoter derived from the Cauliflower Mosaic Virus. BiP ss, signal sequence of BiP2; 4 × Linker, (GGGGS)4; 6 × His, 6 × histidine; HDEL, ER retention sequence; OCS, octopine synthase terminator; LB, left border; RB, right border. The arrows represent the position and direction of the primer pairs used. (**b**) The production yields of the Dup-scFv with or without HDEL depending on growth periods of tobacco plants. Total fresh weight of 3- and 5-week-old tobacco leaves (100 mg) expressing Dup-scFv with or without HDEL were resuspended in 500 μL of buffer, and 5 μl of supernatant proteins were subjected to immunoblotting with anti-HIS antibody. The 15 kDa, 6 × HIS-tagged GFP-nanobody (0 ng, 6.25 ng, 12.5 ng, 25 ng, and 50 ng) was used as standard. (**c**) Protein expression of the Dup-scFv in 5-week-old *N. benthamiana* plants. Total crude proteins were subjected to SDS-PAGE under reducing and non-reducing conditions. Immunoblot analysis was performed using anti-HIS antibody. Ponceau S-stained Rubisco large subunit was used to show the equivalent loading of proteins. (**d**) The protein level of the Dup-scFv with HDEL at each purification step. 10 g of tobacco leaves-expressing Dup-scFv with HDEL were resuspended in 30 mL buffer, and 2.5 μL sample of each step was loaded onto SDS-PAGE gel. The 6 × HIS-tagged nanobody (0 ng, 6.25 ng, 12.5 ng, 25 ng, 50 ng, and 100 ng) was used as standard. Sup, supernatant; Filt, filtrated; F.T, flow-through; W, washing; Elu, elution.

**Figure 2:**
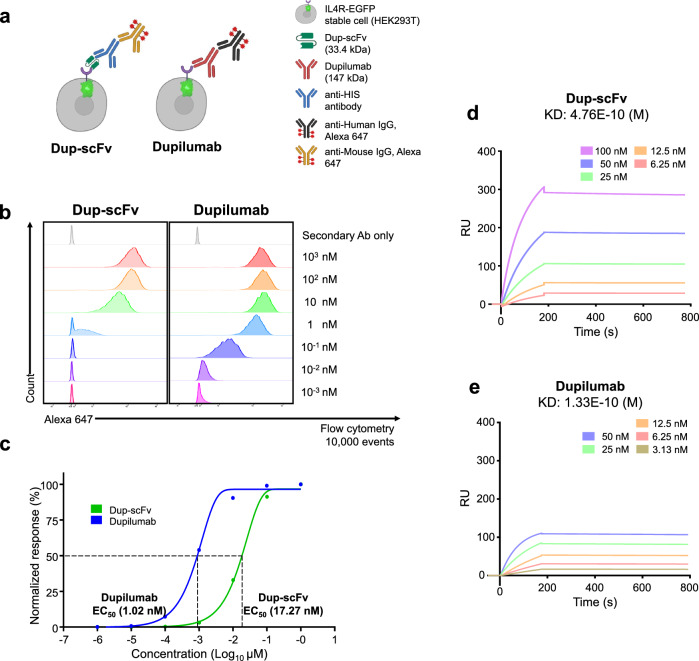
Binding affinity of the Dup-scFv and dupilumab to IL-4Rα (**a**) Schematic representation of the Dup-scFv and dupilumab cell binding. (**b** and **c**) Seven-point curve flow-cytometry analysis (**b**) and normalized response graph **(c)**. Dup-scFv and dupilumab were treated to IL-4Rα overexpressing HEK293T cell. Binding of the Dup-scFv and dupilumab on cells was estimated by adding a secondary antibody labelled with Alexa-647. Untreated cells and cells with only secondary antibody were used as controls. Data were fitted using Graph Pad Prism 8 software. (**d** and **e**) Sensograms for SPR analysis of the Dup-scFv (**d**) and dupilumab (**e**) with immobilized IL-4Rα. The process was done by iMSPR-ProX.

For the successful expression of Dup-scFv in *N. benthamiana*, codon usage optimization was performed, and the sequences of the V_H_ and V_L_ regions of dupilumab were synthesized. Two variants of Dup-scFv were then generated: one has endoplasmic reticulum (ER) signal sequence, HDEL, at the C-terminus of the scFv and the other has no sequences (Fig. 1a). Each construct was co-infiltrated into *N. benthamiana* leaves with p19 using the *Agrobacterium*-mediated method for transient expression. To establish an efficient expression system for Dup-scFv variants in *N. benthamiana*, we evaluated the expression levels of these variants at different stages of tobacco plants, including 3-week-old young and 5-week-old fully expanded mature plants. As shown in Fig. 1b, the expression levels of Dup-scFv variants were not affected by the growth stage of the tobacco plants used. To estimate the expression efficiency of each variant, *N. benthamiana* plants expressing Dup-scFv variants were subjected to immunoblotting. As expected, Dup-scFv with HDEL displayed increased molecular mass owing to the presence of the HDEL tag. The production yields of Dup-scFv with HDEL were approximately 10.1 to 10.5 mg/kg of fresh leaf weight. In contrast, Dup-scFv without HDEL resulted in lower yields, ranging from 1.5 to 1.6 mg/kg. Dup-scFv with HDEL was expressed at approximately 7.0-fold higher levels than Dup-scFv without HDEL (Fig. 1b, 2c, and S2 Table). In addition, we determined the biochemical characteristics of *N. benthamiana*-produced Dup-scFv variant proteins using reducing and non-reducing SDS-PAGE and immunoblot analysis. As a result, these Dup-scFv variants displayed similar migration distances under both reducing and non-reducing conditions, indicating the successful expression of monomeric Dup-scFv molecules (Fig. 1). These results suggest that the ER-retained form, Dup-scFv with HDEL, yielded higher expression; However, the plant growth stages did not affect antibody yields. Considering the protein expression levels of the antibodies, we selected Dup-scFv with HDEL expressed in 5-week-old *N. benthamiana* for further studies. Subsequently, we performed the purification of Dup-scFv with HDEL, monitoring the efficiency of purification and potential loss of Dup-scFv with HDEL protein at each purification step. Protein levels remained consistent after filtering, and no losses were detected at any stage of the purification process (Fig. 1d). According to the standard curve generated using the histidine control, we eluted 10.46 mg (equivalent to 65.4 ng in 2.5 μL, using a total elution volume of 4 mL) from 1 kg of total fresh weight of Dup-scFv with HDEL-expressing tobacco leaves (Data not shown). These results indicate that histidine-tagged Dup-scFv with HDEL was successfully purified using the Ni–NTA resin under these conditions, with no losses during the purification steps.

### Binding affinity and blocking activity of Dup-scFv compared to dupilumab

According to recent reports, as scFv exhibits a broader range of affinity compared to complete immunoglobulins (Ig), confirming the level of affinity of scFv is a crucial initial step for the development of small antibody. Thus, we first determined the binding affinity of Dup-scFv to cells through flow cytometry using an IL4R-EGFP lentiviral transduced cell line in HEK-293 T (Fig. 2a and b). As a result, we observed that the EC_50_ (half maximal effective concentration) value of Dup-scFv was 17.27 nM (Fig. 2c) compare to 1.02 nM of dupilumab, which indicate the cell binding avidity of Dup-scFv was decreased approximately 16.9-fold. Additionally, to further confirm the binding capability of Dup-scFv, we measured the molecular binding kinetics of Dup-scFv via Surface Plasmon Resonance (SPR) analysis and compared it with the binding affinity of dupilumab. As shown in Fig. 2d and e, Dup-scFv (476 pM, d) showed approximately 3.57-fold lower binding affinity than dupilumab (133 pM, e). These results indicate that Dup-scFv has a significant decrease in binding capacity due to the monovalent scFv modification process. However, despite this obvious scFv modification-induced reduction in binding capacity, Dup-scFv still has an excellent pM-level KD as a biologic because dupilumab is an antibody with very high target binding capacity.

**Figure 3:**
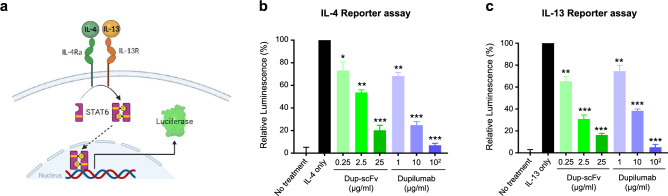
Inhibition of IL-4Ra signaling in reporter assay (**a**) Schematic representation of reporter cell line for tracking IL-4/IL-13 signal cascade. The HEK293 cell line was developed by stable expression of human STAT6 and pSTAT6-induced luciferase. (**b** and **c**) Dose-dependent IL-4/IL-13 signal blocking abilities of dupilumab and the Dup-scFv are measured by luciferase assay. The same concentration (30 ng/mL) of each IL-4 (**b**) and IL-13 (**c**) was used to induce reporter system then serial dose of the Dup-scFv and dupilumab were treated to block the each signaling. Data from four independent experiments are presented as mean ± SEM. *p < 0.05, **p < 0.01, ***p < 0.001 compared between indicated group.

Next, to assess the blocking activity in IL-4/IL-13 cytokine signal transduction by Dup-scFv and dupilumab, we established a reporter cell line expressing luciferase through STAT6 phosphorylation (Fig. 3a). IL-4 or IL-13 were added to Cells, which were pre-incubated with Dup-scFv or dupilumab at the same molar ratio, and the luciferase signals were observed. Figure 3b showed that IL-4-induced luciferase signals were reduced by 26.9, 46.2, and 79.8% in the presence of 0.25, 2.5, and 25 μg/ml of Dup-scFv treatment, respectively. In the presence of IL-13 treatment, luciferase signaling was also significantly reduced in a Dup-scFv concentration-dependent manner, showing a very similar pattern to that observed with dupilumab treatment (Fig. 3). These results indicate that Dup-scFv efficiently masks the IL-4/IL-13-induced cytokine signaling transduction as a functional antibody.

### Dup-scFv treatment on human nasal epithelial cells and its blocking activity through marker genes

**Figure 4:**
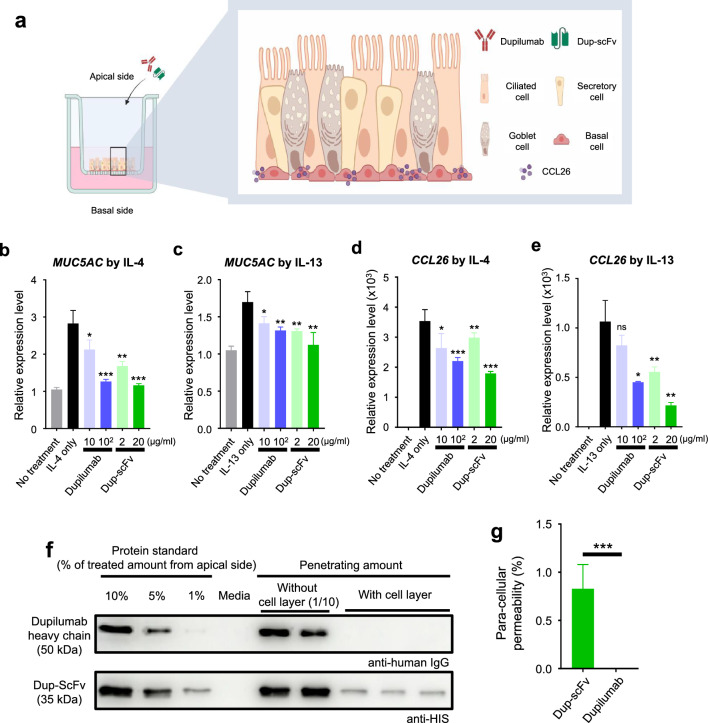
Paracellular permeability of Dup-scFv on the human nasal epithelial cell and its signal blocking activity (**a**) Schematic representation of experiment for evaluating paracellular permeability of Dup-scFv and dupilumab with simplified composition of the HNECs. Dup-scFv and dupilumab were directly treated on the apical side, and 20 ng/mL of IL-4/IL-13 were treated in culture medium on the basal side. (**b**–**e**) Expression of *MUC5AC* and *CCL26* with administration of both Dup-scFv and dupilumab in 20 ng/mL of IL-4 (**b, d**) and IL-13 (**c, e**) supplemented condition in HNECs were detected by quantitative PCR. 3 independent experiments were performed in each experiment. Data are presented as mean ± SEM. *p < 0.05, **p < 0.01, ***p < 0.001, ns; not significant, compared to IL-4 or IL-13 treated condition of each experiment. (**f**) Permeability of Dup-ScFv through HNEC is confirmed by Western blotting. Two The signature cytokines IL-4/IL-13 (20 ng/mL) are treated in the culture medium on the basal side of HNECs for 24 h. (**g**) 5 independent experiments were summarized for permeability test calculation. Data are presented as mean ± SEM. ***p < 0.001.

The paracellular permeability to access deeper part of cells was tested by in air–liquid interface cultured HNECs using trans-well chambers after air side administration of Dup-scFv and dupilumab (Fig. 4a). The receptor-blocking function was determined based on the expression levels of two marker genes, *MUC5AC* and *CCL26*, which are known to be upregulated by IL-4 (Fig. 4b, d) and IL-13 (Fig. 4c, e) treatment. The expression level of *MUC5AC* was increased by 2.8-fold and 1.7-fold under IL-4 and IL-13 treatment, respectively. These increased mRNA levels were almost restored to base line4 by treatment of Dup-scFv (20 μg/mL, 598.8 nM) or dupilumab (100 μg/mL, 680.27 nM). The receptor-blocking effect of Dup-scFv and dupilumab was obvious, but they did not show significantly difference (Fig. 4b, c). In case of *CCL26*, the expression was significantly increased by more than 3000-fold and 1000-fold in response to IL-4 and IL-13, respectively. The IL-4-induced expression levels were decreased to 2200-fold and 1790-fold by the additional treatment of dupilumab (100 μg/ml) or Dup-scFv (20 μg/ml), respectively. In addition, the IL-13-induced *CCL26* expression level was reduced by 57.6% and 79.7% in response to dupilumab (100 μg/ml) or Dup-scFv (20 μg/mL) (Fig. 4f, g), respectively. These data indicate that the expression of CCL26, expressed on basal cells located in the deep part of the HNECs layer cultured in transwells, was more efficiently controlled by Dup-scFv at all doses. Next, the level of paracellular permeability of dupilumab and Dup-scFv was assessed by measuring the signal derived from the protein bands on a Western blot (Fig. 4f, g). Immunoblotting the culture medium from lower chamber indicate the amount of each antibody that passed through the HNEC layer for 48 h (Fig. 4f, g). As a result, no signals of dupilumab were detected, while approximately 0.8% of Dup-scFv was detected in the basal side culture medium. These results suggest that, unlike dupilumab, Dup-scFv seems to pass the HNECs layer cultured in transwell. Collectively, our data demonstrated that Dup-scFv, which exhibits paracellular permeability, is more effective compared to dupilumab in blocking receptors located deep in the cell layer.

## Discussion

The advantages of producing scFv forms that can be generated in a single chain have long attracted attention in the field of biologics, particularly plant-based biotechnology^24^. In addition to scFv antibodies against plant pathogens, scFvs against botulinum neurotoxin A (BoNT/A), human epidermal growth factor receptor 1 (HER1), HER2, and Gibberlin (a peptide hormone inhibiting appetite) have been produced in live *N. Benthamiana*, *N. tabacum*, and *A. thaliana*, among other plants^25,26^. However, none of the plant-derived scFvs developed as vaccines, targeted anticancer drugs, or antidotes have advanced to clinical trials. Meanwhile, small modified antibodies, including nanobodies and scFvs, have been applied in various diagnostic imaging tools and radioisotope-conjugated ADCs (antibody–drug-conjugates) for the diagnosis of solid tumors due to their tissue penetrating ability^27,28^. Despite some reports of tissue penetrating properties, none of these have been developed as non-invasive medicines^29,30^.

In this study, we report that plant-based Dup-scFv efficiently inhibits the inflammatory response of HNECs cultured at air–liquid interface, not only by preserving the binding function of the parent antibody, but also by its high tissue penetration due to its very small size compared to the parent antibody. In general, scFv variants exhibit approximately 100- to 500-fold reduced binding affinity compared to full-length parental mAbs^31^. Dup-scFv, on the other hand, remains relatively close to its original binding affinity. Dup-svFv shows some reduction in binding strength, with an EC_50_ about 17-fold lower than the original dupilumab and a KD about 3.5-fold higher, but retains most of the high binding capacity of the original antibody. On the other hand, the permeability of a protein through epithelial cells is entirely dependent on the size of the protein^32^. Western blots analyzing the amount of air-side-treated dupilumab and Dup-scFv passing through the cell layer of air–liquid interface cultured HNECs and found in the underlying media showed that only Dup-scFv passes through the HNECs layer and moves about 4% of the total amount treated into the underlying media (Fig. 4f). Furthermore, Dup-scFv significantly inhibited *CCL26* inflammatory marker expression in basal cells, which are hardly accessible from the air side, more effectively than dupilumab. It has been reported that in patients with chronic rhinosinusitis, the expression of proteins involved in intercellular aggregation, such as *ZO-1*, *claudin*, and *occludin*, is significantly decreased, resulting in increased intracellular gaps between epithelial cells and thus increased material permeability^20,33^. Taken together, these facts and our results suggest that Dup-scFv's high tissue permeability and inflammation inhibition ability due to its small size may further suggest its therapeutic potential by non-invasive nasal administration.

In this study, we tried to obtain the high yield of expression of dupilumab-scFv in N. benthamiana via the codon optimization for appropriate codon usage in plants and ER-retention to increase protein stability. However, the protein expression yield of dupilumab-scFv in N. benthamiana is quite low. Thus, further research is required to enhance the yield. Recently, the possibility has been proposed that the expression yields of plant-based protein pharmaceuticals can be greatly increased by newly developed vector systems, such as MagnoCon^®^, and infiltration methods applied to the entire tobacco leaf through a vacuum chamber. Especially, it has been reported that the MagniCon^®^ vector system significantly promotes the transient expression of exogenous proteins by introducing the characteristics of plant viruses, which suppress the expression of endogenous plant protein and overexpress the viral proteins^4^. Despite the efficiency of MagniCon^®^ vector system, researchers are not able to easily utilize it due to the high royalty. Thus, the development of efficient vector systems for high expression yields is an important aim of current research in plant-based protein pharmaceuticals. In this study, we suggested the possibility that dupilumab-scFv expressed in *N. benthamiana* also possesses high therapeutic efficacy. Therefore, if the expression yields of plant-based pharmaceuticals are improved, we cautiously propose that plant-expressed dupilumab-scFv could be utilized as a strong candidate for industrial application.

Developing plant-derived medicines that retain the benefits of plant proteins but without the potential risks is a promising direction in plant biotechnology. For conditions such as allergies, which affect about 30% of the world's population and are considered a modern-day epidemic, there is a steadily increasing demand for various non-invasive treatments^34,35^. Because these diseases are common, non-life-threatening conditions, injections are burdensome, so there is more room to consider protein host preferences in biopharmaceuticals, and non-invasive plant biologics are likely to be favored.

In this study, we demonstrated that scFv derived from dupilumab, which has shown high therapeutic efficacy in localized immune diseases such as allergy, was produced in plants and applied non-invasively to effectively suppress inflammation in human nasal epithelial cells cultured at the air–liquid interface. These results suggest the potential of plant-based small antibodies as a non-invasive route of treatment for autoimmune diseases.

## Material & methods

### Construction of the dupilumab-scFv expression vector in *N. benthamiana*

The sequences of the heavy and the light chains of dupilumab were extracted from DrugBank (https://go.drugbank.com/drugs/DB12159). The Fab parts of the heavy and light chains were connected with 4 × G4S linkers. In addition, the 6 × HIS tag was connected before the stop codon to enable purification. For ER localization, the signal sequence of *Arabidopsis thaliana* luminal binding protein (AtBiP2 ss) and the ER retention signal sequence, HDEL, were inserted into the N-terminal and C-terminal regions, respectively. The final amino acid sequence was codon-optimized for high-yield plant expression of Dup-scFv. The DNA and amino acid sequences are shown in S1 and S2 Figs. Plant codon-optimized Dup-scFv DNA sequences were cloned into a pEarlyGate 100 binary vector (*p35S:AtBiP2 ss-plant codon-optimized Dup-scFv* and *p35S:AtBiP2 ss-plant codon-optimized Dup-scFv-HDEL*). The resulting constructs were co-transformed into *Agrobacterium tumefaciens* GV3101 competent cells using the freeze–thaw method. Transformed agrobacteria were incubated in YEB liquid medium containing 50 μg/ml kanamycin and 50 μg/ml rifampicin at 28 °C for 16 h. Agrobacteria were resuspended in infiltration solution (10 mM MES, pH 5.7, 10 mM MgCl_2_, and 500 μM acetosyringone) and infiltrated into the abaxial side of *N. benthamiana* leaves, aged 3–5 weeks, using a syringe as described in^36^. The expressions of AtBiP2 ss-plant codon-optimized Dup-scFv and AtBiP2 ss-plant codon-optimized Dup-scFv-HDEL were enhanced by co-expressing the virus-encoded silencing suppressor, P19, which enables high levels of transient expression^37^. After 2–3 days of incubation, the infiltrated leaves were harvested for all experiments.

### Purification of plant-produced Dup-scFv from *N.benthamiana* leaves

Infiltrated *N.benthamiana* leaves were ground in liquid nitrogen using a mortar and pestle to extract crude proteins. The powder was suspended in two volumes of protein extraction buffer (1 × PBS, 300 mM NaCl, 10 mM imidazole, and protease inhibitor cocktail [Abbkine, BMP1001]) and incubated at 4 °C for 1 h. The protein suspensions were centrifuged at 15,000 rpm for 20 min at 4 °C. The supernatant was filtered through Miracloth to remove debris and then loaded onto the Ni–NTA agarose resin (Qiagen, 30,230). The column was washed with the washing buffer (1 × PBS, 300 mM NaCl, and 20 mM imidazole), and scFv was eluted using elution buffer (1 × PBS, 300 mM NaCl, and 300 mM imidazole). After elution, the Silde-A-Lyzer Dialysis Casette (Thermo Fisher Scientific, 66,380) and the Amicon Ultra Centrifugal filter (Sigma-Aldrich, UFC901008) were used to concentrate the purified Dup-scFv protein and remove imidazole. Purified Dup-scFv was stored at − 80 °C.

### Cells

The HEK293T and HEK293 cell lines were purchased from the Korea Cell Line Bank. The cells were cultured in DMEM high glucose media (Gibco, 11,995–065) supplemented with 10% FBS (Gibco, 26,140–079) and penicillin/streptomycin (Gibco, 15,140–122) at 37 °C with 5% CO_2_.

### Flow cytometry IL-4Rα cell binding assay

Human IL-4Rα-overexpressing HEK293T cells were seeded at ${2\times 10}^{5}$ cell/tube in a PCR tube containing 100 µl PBS. The cells were collected, washed in PBS, and incubated with Dup-scFv or dupilumab. After binding to Dup-scFv, the cells underwent a washing process, followed by exposure to the 6xHIS tag monoclonal antibody (Thermo Fisher Scientific, MA1-21,315). The cells were then washed and incubated for 15 min with Alexa Fluor-647 AffiniPure Goat anti-mouse IgG (H + L) (Jackson ImmunoResearch, 115-605-003) and Alexa Fluor-647 AffiniPure Goat anti-human IgG (H + L) (Jackson ImmunoResearch, 109-605-003), respectively. Finally, the cells were subjected to flow cytometer (BD FACSLyric). The EC_50_ and binding affinity data were analyzed using Graph Pad Prism 8 and FlowJo software v10.

### Surface plasmon resonance (SPR)

Surface plasmon resonance (SPR) was performed using an iMSPR-ProX. Biotinylated IL-4Rα was immobilized on the sensor chip (A-Dex100, DCAV1100) at a capture level of 169.7 response units (RUs). The binding analysis was conducted using seven concentrations of Dup-scFv: 1.56, 3.125, 6.25, 12.5, 25, 50, and 100 nM with a flow rate of 100 μl/ml. Regeneration of the sensor chip was performed with regeneration buffer (10 mM glycine–HCl, pH 1.5) with 100 μl/ml of flow rate. To compute the equilibrium dissociation rate constants (k_a_, k_d_, and k_D_), the 1:1 kinetic binding model was used.

### STAT6 reporter assay for measuring IL-4Rα inhibition

Human STAT6 based on the puromycin-resistant lentivirus system and pSTAT6-induced luciferase based on the blasticidin-resistant system were purchased from Addgene (#81,950 and #35,554, respectively) for lentiviral transduction into the HEK293 cell line. Before the experiment, ${5\times 10}^{4}$ of reporter cells were seeded into each well of a white 96-well plate. Cells were treated with Dup-scFv or dupilumab in the presence of DMEM for 1 h at 37 °C and 5% CO_2_. Thereafter, IL-4 (Enzynomics, C008) and IL-13 (Enzynomics, C009) were added to each well and incubated in DMEM for 24 h at 37 °C and 5% CO_2_. After removing the medium, the cells were resuspended and washed with PBS, and lysed with 20 µl of lysis buffer (Promega, E153A). Random luminescence unit was measured using a Centro XS3 luminometer (EG&G Berthold, LB960) after the addition of 50 µl of luciferase substrate (Promega, E1501). The cytokine concentrations used were 30 ng/ml of IL-4 and 30 ng/ml of IL-13.

### Paracellular permeability analysis

This study was approved by the Institutional Review Board of Yonsei University College of Medicine (4-2016-1153) that all methods were performed in accordance with the relevant guidelines and regulations. Informed written consent was obtained from participants who recruited between October 2021 and February 2022, and for pediatric patients under the age of 19, informed consent was obtained from parents after full explanation. To assess paracellular permeability, fully differentiated HNECs were used for the experiment after establishment of the air–liquid interface (ALI), using specimen from surgery for chronic rhinosinusitis with polyps as previously reported^20^. Dup-scFv and dupilumab were treated together on the apical side of the HNECs with each concentration of 50 μg/mL and 100 μg/mL, respectively, in 100 μL of media. In addition, 20 ng/μL of IL-4/IL-13 was added to the medium on the basal side. After 48 h of incubation at 37 °C and 5% CO_2_, the culture media were subjected to western blot analysis. Mouse anti-His-tag monoclonal antibody (Invitrogen, MA1-21,315) was used as the primary antibody for detecting Dup-scFv. Goat anti-mouse IgG-HRP (Jackson ImmunoResearch, 115-034-003) and goat anti-human IgG-HRP (Jackson ImmunoResearch, 109-035-003) were used as secondary antibodies to measure the levels of Dup-scFv and dupilumab, respectively.

### qPCR analysis of inflammatory marker genes in HNECs

The IL4 receptor-blocking function of Dup-scFv and dupilumab via topical administration on the apical side of HNECs were determined by qPCR analysis. The expression levels of two marker genes, *MUC5AC* and *CCL26* were used to measure the function after 20 ng/mL of the IL-4/IL-13 treated in culture medium on the basal side. Total RNA was extracted from the HNECs using Trizol (Favorgen, FATRR001). The extract was then subjected to phase separation via the addition of chloroform (Sigma-Aldrich, C2432). The upper aqueous phase was carefully collected, and total RNA was then precipitated using isopropanol. The isolated total RNA was reverse transcribed using the SuperiorScript III reverse transcriptase kit (Enzynomics, RT006). For quantitative PCR, the QuantStudio 3 Real-time PCR instrument (Applied Biosystems, A28132) was used in combination with the AccuPower^®^ 2X Greenstar qPCR master mix (Bioneer, K-6251). The specific sequences of the PCR primers used in this study can be found in the supplementary materials.

### Statistical analysis

Statistical analyses were performed using GraphPad Prism 8 (GraphPad Software) with an unpaired Student’s *t* test as indicated in the figure legends. Data are presented as mean ± SD and *p < 0.05, ** *p* < 0.01, ***p < 0.001, and ****p < 0.0001 were considered to indicate statistical significance. A nonlinear regression curve was constructed by using log versus response with a three-parameter function.

## Regulatory and compliance

Experiments on plants in this work comply with the IUCN Policy Statement on Research Involving Species at Risk of Extinction and the Convention on the Trade in Endangered Species of Wild Fauna and Flora. The plants (*Nicotiana benthamiana*) and studies using these plants were approved by the Biosafety Committee of Yonsei University. Seeds and plants used in the experiments are not listed as threatened and were obtained from the publicly available seed company.

### Supplementary Information

Supplementary Information.

## Data Availability

The datasets generated and/or analyzed during the current study are available in the Genebank. [Dup_scFv_HDEL; PP711326, Dup_scFv; PP711327].
