# Supplementary material for: Nicotiana benthamiana-derived dupilumab-scFv reaches deep into the cultured human nasal epithelial cells and inhibits CCL26 expression
Source: Sci Rep. 2024 Jun 24;14:14558. doi: 10.1038/s41598-024-65524-0 (PMC11196582; doi:10.1038/s41598-024-65524-0)
Supplement: Supplementary file 1 — Supplementary Information. [file 41598_2024_65524_MOESM1_ESM.pptx]

## Slide 1
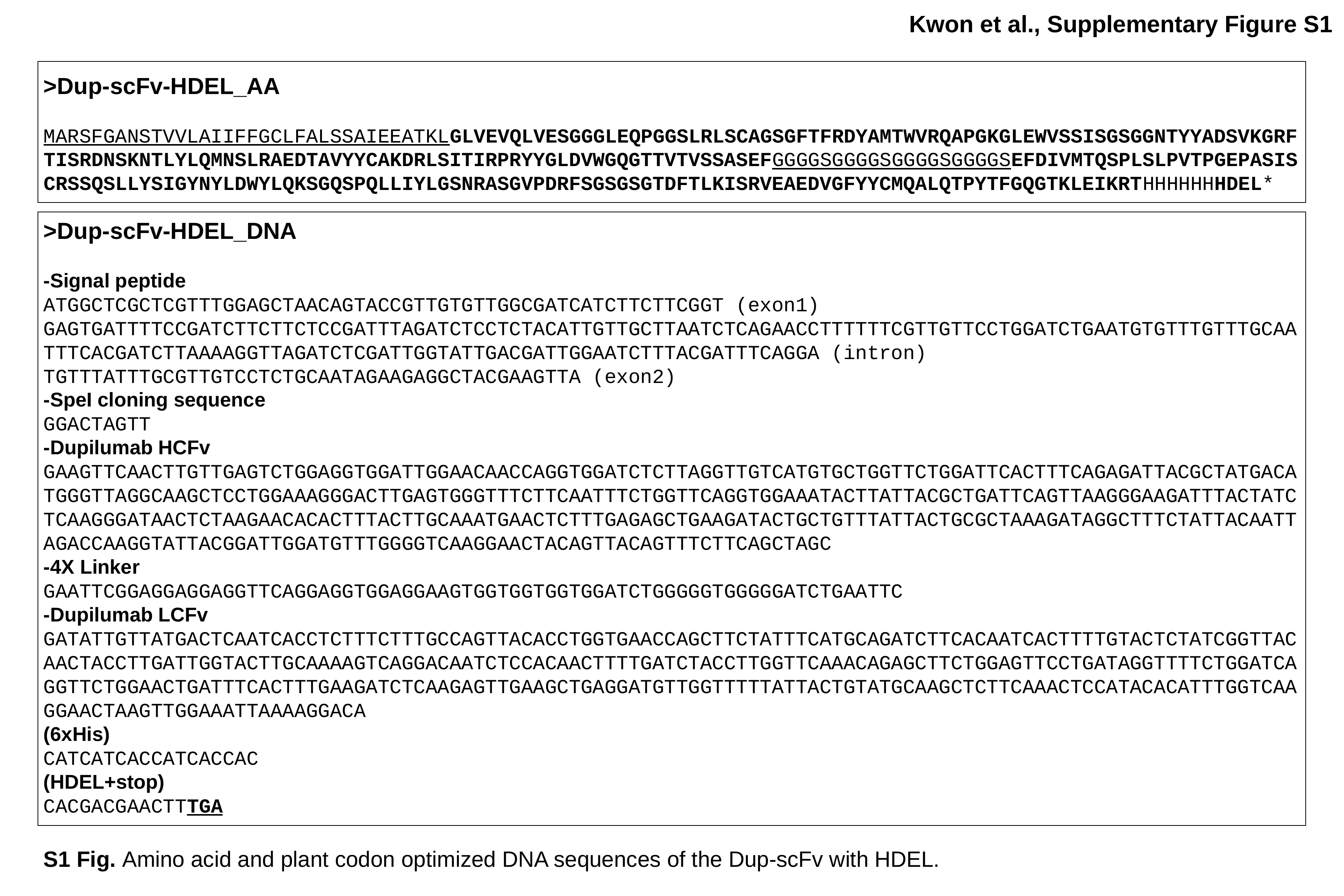

Kwon et al., Supplementary Figure S1
>Dup-scFv-HDEL_AA
MARSFGANSTVVLAIIFFGCLFALSSAIEEATKLGLVEVQLVESGGGLEQPGGSLRLSCAGSGFTFRDYAMTWVRQAPGKGLEWVSSISGSGGNTYYADSVKGRFTISRDNSKNTLYLQMNSLRAEDTAVYYCAKDRLSITIRPRYYGLDVWGQGTTVTVSSASEFGGGGSGGGGSGGGGSGGGGSEFDIVMTQSPLSLPVTPGEPASISCRSSQSLLYSIGYNYLDWYLQKSGQSPQLLIYLGSNRASGVPDRFSGSGSGTDFTLKISRVEAEDVGFYYCMQALQTPYTFGQGTKLEIKRTHHHHHHHDEL*
>Dup-scFv-HDEL_DNA
-Signal peptide
ATGGCTCGCTCGTTTGGAGCTAACAGTACCGTTGTGTTGGCGATCATCTTCTTCGGT (exon1)
GAGTGATTTTCCGATCTTCTTCTCCGATTTAGATCTCCTCTACATTGTTGCTTAATCTCAGAACCTTTTTTCGTTGTTCCTGGATCTGAATGTGTTTGTTTGCAATTTCACGATCTTAAAAGGTTAGATCTCGATTGGTATTGACGATTGGAATCTTTACGATTTCAGGA (intron)
TGTTTATTTGCGTTGTCCTCTGCAATAGAAGAGGCTACGAAGTTA (exon2)
-SpeI cloning sequence
GGACTAGTT
-Dupilumab HCFv
GAAGTTCAACTTGTTGAGTCTGGAGGTGGATTGGAACAACCAGGTGGATCTCTTAGGTTGTCATGTGCTGGTTCTGGATTCACTTTCAGAGATTACGCTATGACATGGGTTAGGCAAGCTCCTGGAAAGGGACTTGAGTGGGTTTCTTCAATTTCTGGTTCAGGTGGAAATACTTATTACGCTGATTCAGTTAAGGGAAGATTTACTATCTCAAGGGATAACTCTAAGAACACACTTTACTTGCAAATGAACTCTTTGAGAGCTGAAGATACTGCTGTTTATTACTGCGCTAAAGATAGGCTTTCTATTACAATTAGACCAAGGTATTACGGATTGGATGTTTGGGGTCAAGGAACTACAGTTACAGTTTCTTCAGCTAGC
-4X Linker
GAATTCGGAGGAGGAGGTTCAGGAGGTGGAGGAAGTGGTGGTGGTGGATCTGGGGGTGGGGGATCTGAATTC
-Dupilumab LCFv
GATATTGTTATGACTCAATCACCTCTTTCTTTGCCAGTTACACCTGGTGAACCAGCTTCTATTTCATGCAGATCTTCACAATCACTTTTGTACTCTATCGGTTACAACTACCTTGATTGGTACTTGCAAAAGTCAGGACAATCTCCACAACTTTTGATCTACCTTGGTTCAAACAGAGCTTCTGGAGTTCCTGATAGGTTTTCTGGATCAGGTTCTGGAACTGATTTCACTTTGAAGATCTCAAGAGTTGAAGCTGAGGATGTTGGTTTTTATTACTGTATGCAAGCTCTTCAAACTCCATACACATTTGGTCAAGGAACTAAGTTGGAAATTAAAAGGACA
(6xHis)
CATCATCACCATCACCAC
(HDEL+stop)
CACGACGAACTTTGA
S1 Fig. Amino acid and plant codon optimized DNA sequences of the Dup-scFv with HDEL.

## Slide 2
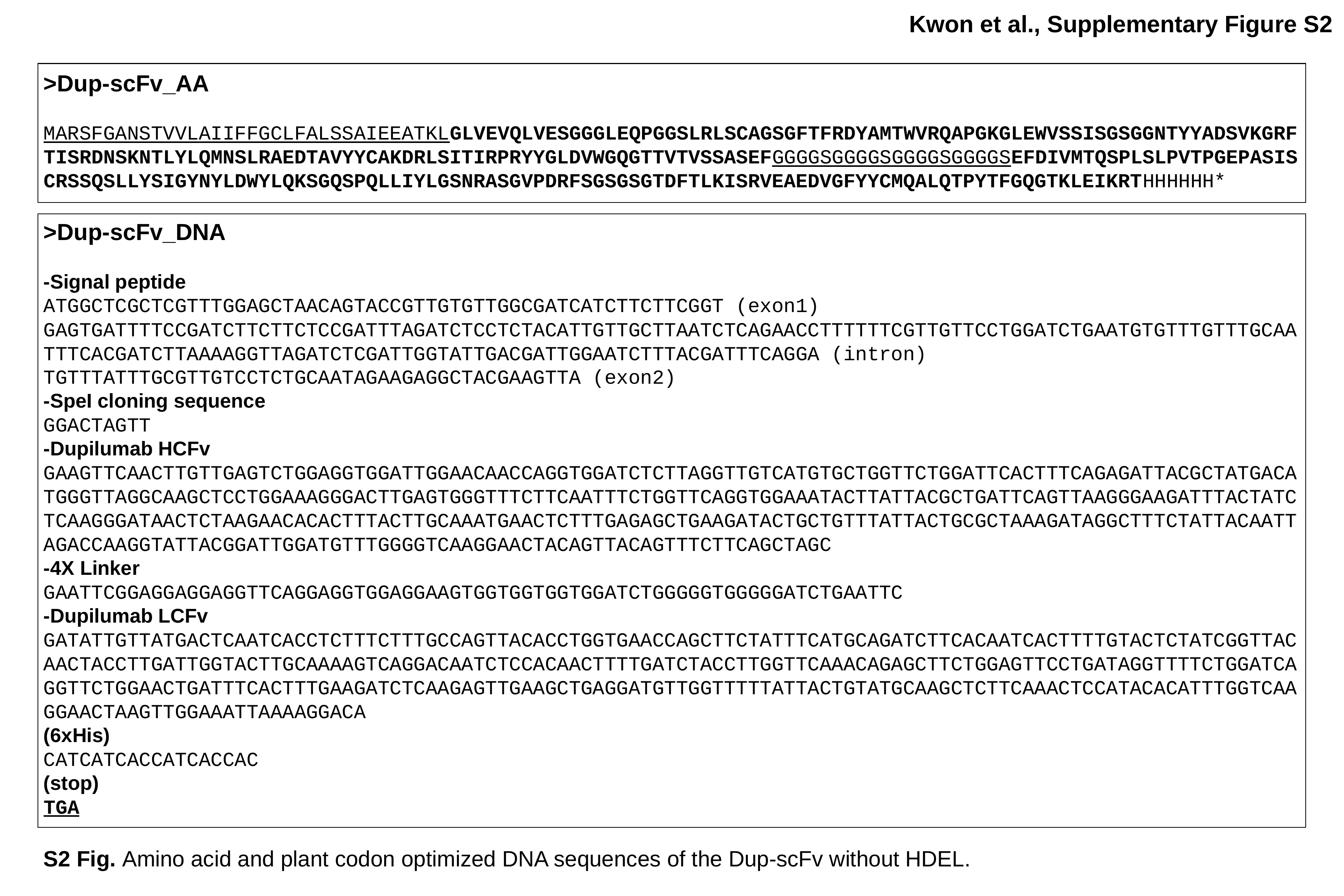

Kwon et al., Supplementary Figure S2
>Dup-scFv_AA
MARSFGANSTVVLAIIFFGCLFALSSAIEEATKLGLVEVQLVESGGGLEQPGGSLRLSCAGSGFTFRDYAMTWVRQAPGKGLEWVSSISGSGGNTYYADSVKGRFTISRDNSKNTLYLQMNSLRAEDTAVYYCAKDRLSITIRPRYYGLDVWGQGTTVTVSSASEFGGGGSGGGGSGGGGSGGGGSEFDIVMTQSPLSLPVTPGEPASISCRSSQSLLYSIGYNYLDWYLQKSGQSPQLLIYLGSNRASGVPDRFSGSGSGTDFTLKISRVEAEDVGFYYCMQALQTPYTFGQGTKLEIKRTHHHHHH*
>Dup-scFv_DNA
-Signal peptide
ATGGCTCGCTCGTTTGGAGCTAACAGTACCGTTGTGTTGGCGATCATCTTCTTCGGT (exon1)
GAGTGATTTTCCGATCTTCTTCTCCGATTTAGATCTCCTCTACATTGTTGCTTAATCTCAGAACCTTTTTTCGTTGTTCCTGGATCTGAATGTGTTTGTTTGCAATTTCACGATCTTAAAAGGTTAGATCTCGATTGGTATTGACGATTGGAATCTTTACGATTTCAGGA (intron)
TGTTTATTTGCGTTGTCCTCTGCAATAGAAGAGGCTACGAAGTTA (exon2)
-SpeI cloning sequence
GGACTAGTT
-Dupilumab HCFv
GAAGTTCAACTTGTTGAGTCTGGAGGTGGATTGGAACAACCAGGTGGATCTCTTAGGTTGTCATGTGCTGGTTCTGGATTCACTTTCAGAGATTACGCTATGACATGGGTTAGGCAAGCTCCTGGAAAGGGACTTGAGTGGGTTTCTTCAATTTCTGGTTCAGGTGGAAATACTTATTACGCTGATTCAGTTAAGGGAAGATTTACTATCTCAAGGGATAACTCTAAGAACACACTTTACTTGCAAATGAACTCTTTGAGAGCTGAAGATACTGCTGTTTATTACTGCGCTAAAGATAGGCTTTCTATTACAATTAGACCAAGGTATTACGGATTGGATGTTTGGGGTCAAGGAACTACAGTTACAGTTTCTTCAGCTAGC
-4X Linker
GAATTCGGAGGAGGAGGTTCAGGAGGTGGAGGAAGTGGTGGTGGTGGATCTGGGGGTGGGGGATCTGAATTC
-Dupilumab LCFv
GATATTGTTATGACTCAATCACCTCTTTCTTTGCCAGTTACACCTGGTGAACCAGCTTCTATTTCATGCAGATCTTCACAATCACTTTTGTACTCTATCGGTTACAACTACCTTGATTGGTACTTGCAAAAGTCAGGACAATCTCCACAACTTTTGATCTACCTTGGTTCAAACAGAGCTTCTGGAGTTCCTGATAGGTTTTCTGGATCAGGTTCTGGAACTGATTTCACTTTGAAGATCTCAAGAGTTGAAGCTGAGGATGTTGGTTTTTATTACTGTATGCAAGCTCTTCAAACTCCATACACATTTGGTCAAGGAACTAAGTTGGAAATTAAAAGGACA
(6xHis)
CATCATCACCATCACCAC
(stop)
TGA
S2 Fig. Amino acid and plant codon optimized DNA sequences of the Dup-scFv without HDEL.

## Slide 3
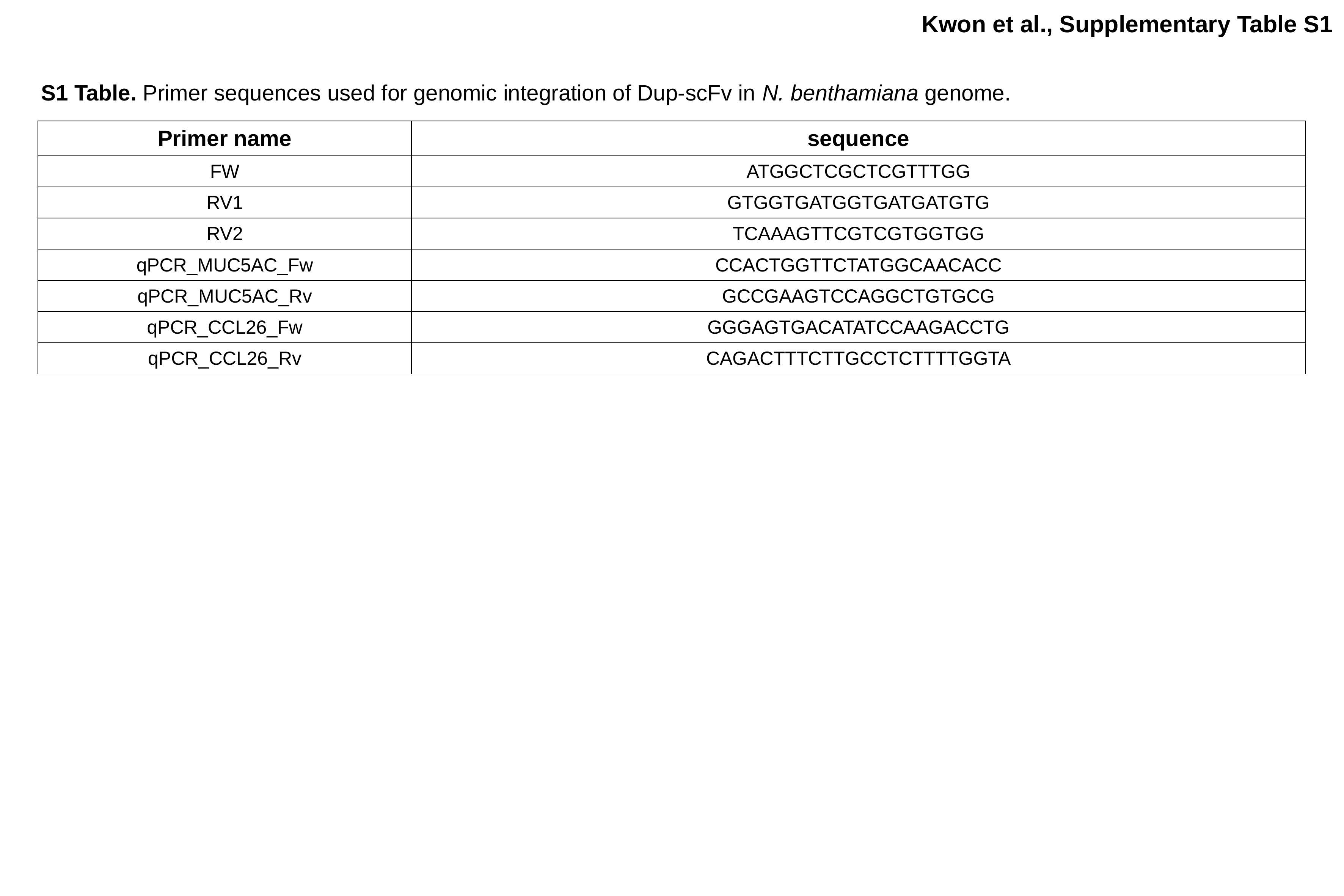

Kwon et al., Supplementary Table S1
S1 Table. Primer sequences used for genomic integration of Dup-scFv in N. benthamiana genome.
| Primer name | sequence |
| --- | --- |
| FW | ATGGCTCGCTCGTTTGG |
| RV1 | GTGGTGATGGTGATGATGTG |
| RV2 | TCAAAGTTCGTCGTGGTGG |
| qPCR\_MUC5AC\_Fw | CCACTGGTTCTATGGCAACACC |
| qPCR\_MUC5AC\_Rv | GCCGAAGTCCAGGCTGTGCG |
| qPCR\_CCL26\_Fw | GGGAGTGACATATCCAAGACCTG |
| qPCR\_CCL26\_Rv | CAGACTTTCTTGCCTCTTTTGGTA |

## Slide 4
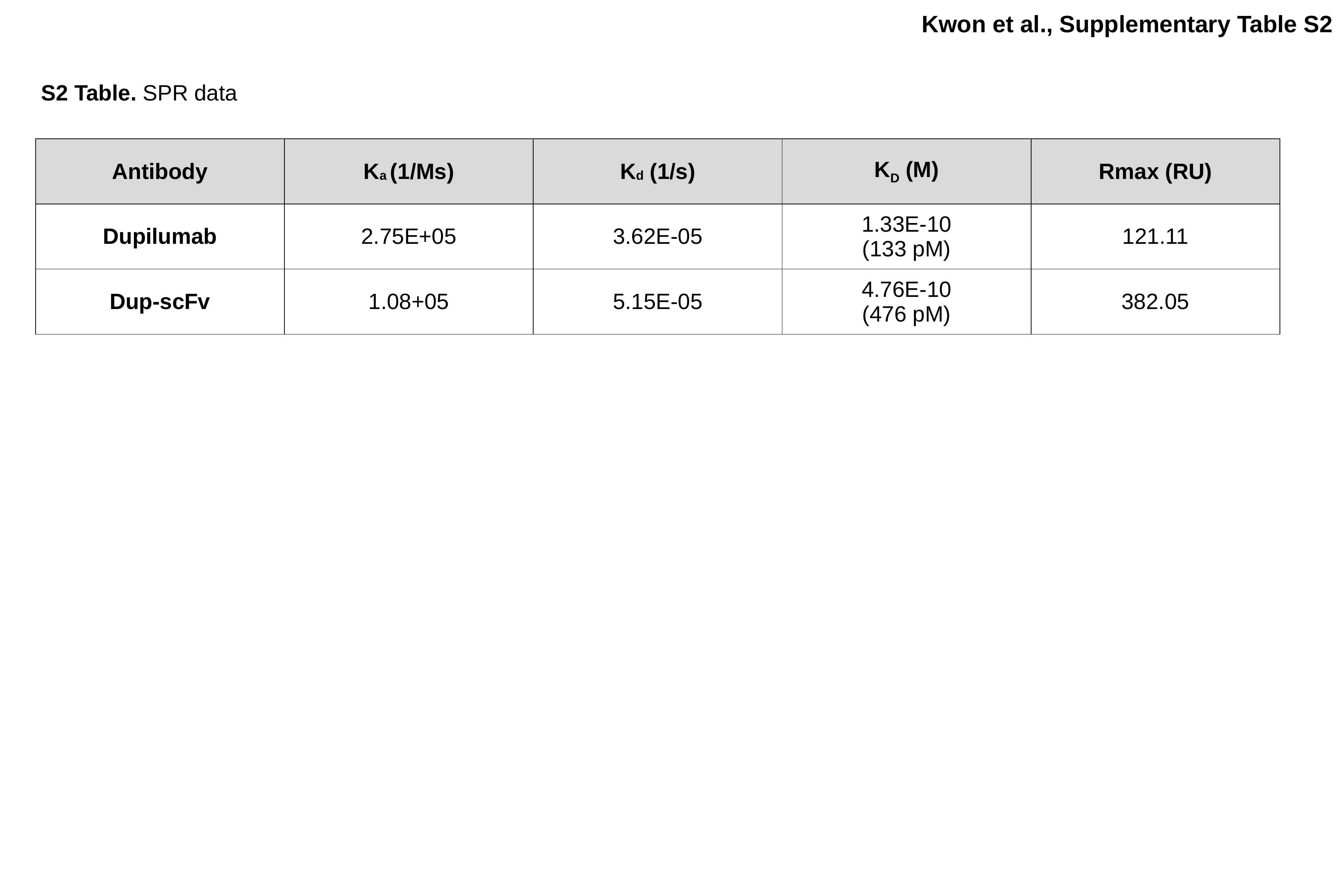

Kwon et al., Supplementary Table S2
S2 Table. SPR data
| Antibody | Ka (1/Ms) | Kd (1/s) | KD (M) | Rmax (RU) |
| --- | --- | --- | --- | --- |
| Dupilumab | 2.75E+05 | 3.62E-05 | 1.33E-10 (133 pM) | 121.11 |
| Dup-scFv | 1.08+05 | 5.15E-05 | 4.76E-10 (476 pM) | 382.05 |
